# Socio-ecological dynamics and challenges to the governance of Neglected Tropical Disease control

**DOI:** 10.1186/s40249-016-0235-5

**Published:** 2017-02-06

**Authors:** Edwin Michael, Shirin Madon

**Affiliations:** 1 Eck Institute for Global Health, Department of Biological Sciences, University of Notre Dame, Notre Dame, USA; 2 Department of International Development, London School of Economics and Political Science, London, UK; 3 Department of Management, London School of Economics and Political Science, London, UK

**Keywords:** Neglected tropical diseases, Lymphatic filariasis, Schistosomiasis, Malaria, Smallpox, Socio-ecological dynamics, Complex dynamical systems, Normal science paradigm, Organizational theory, Complexity-based governance

## Abstract

The current global attempts to control the so-called “Neglected Tropical Diseases (NTDs)” have the potential to significantly reduce the morbidity suffered by some of the world’s poorest communities. However, the governance of these control programmes is driven by a managerial rationality that assumes predictability of proposed interventions, and which thus primarily seeks to improve the cost-effectiveness of implementation by measuring performance in terms of pre-determined outputs. Here, we argue that this approach has reinforced the narrow normal-science model for controlling parasitic diseases, and in doing so fails to address the complex dynamics, uncertainty and socio-ecological context-specificity that invariably underlie parasite transmission. We suggest that a new governance approach is required that draws on a combination of non-equilibrium thinking about the operation of complex, adaptive, systems from the natural sciences and constructivist social science perspectives that view the accumulation of scientific knowledge as contingent on historical interests and norms, if more effective control approaches sufficiently sensitive to local disease contexts are to be devised, applied and managed. At the core of this approach is an emphasis on the need for a process that assists with the inclusion of diverse perspectives, social learning and deliberation, and a reflexive approach to addressing system complexity and incertitude, while balancing this flexibility with stability-focused structures. We derive and discuss a possible governance framework and outline an organizational structure that could be used to effectively deal with the complexity of accomplishing global NTD control. We also point to examples of complexity-based management structures that have been used in parasite control previously, which could serve as practical templates for developing similar governance structures to better manage global NTD control. Our results hold important wider implications for global health policy aiming to effectively control and eradicate parasitic diseases across the world.

**Electronic supplementary material:**

The online version of this article (doi:10.1186/s40249-016-0235-5) contains supplementary material, which is available to authorized users.

## Multilingual abstracts

Please see Additional file 1 for translations of the abstract into the six official working languages of the United Nations.

## Introduction

The emerging global initiatives to control the “Neglected Tropical Diseases (NTDs)” have been described on one hand as a major humanitarian effort to reduce the disease burdens of the marginalized populations of the global South [1–3]. In this regard, advocates have used social justice and equity but also political economy arguments to signify that investments in NTD control based on mass drug administration (MDA) are pro-poor, cost-effective and welfare improving. Indeed, the current initiative is promoted as a comprehensive blueprint towards “rescuing the bottom billion from poverty” [4]. On the other hand, the NTD strategy, like other global intervention programmes, such as those against HIV/AIDs, tuberculosis and malaria, also reflects the now long established paradigm in conducting and delivering global health interventions, *viz.* the transnational nature of health technology delivery in poorer countries whereby decision making and power are shifted away from national governments to globally interrelated “epistemic communities” that frame problems, generate knowledge and solutions, and develop advocacy and funding strategies for taking action [5–8]. These characteristics of global health intervention programmes, particularly their narrowly conceptualized focus on therapeutic access and dependence on an extensive external/global locus for problem formulation and solution, have thus come to underpin the features increasingly resembling public health delivery in poorer countries of the global South, particularly in the case of Africa: 1) a hollowing out of state power, 2) a fragmentation of national publics into “communities” gathered around specific needs leading to the “projectification’ of health care focused on specific diseases and islands of intervention, 3) the enactment of standardized, increasingly pharmaceutical-driven biomedical interventions based on narrow worldviews and perspectives emphasizing individual risk factors, and 4) the increasing propensities of such biomedical campaigns for controlling bodies and populations through forms of prescribed management and administration aimed at cultivating passive accepting publics [5–8]. While the study of these biopolitical outcomes has so far focused on the tensions that arise between powerful global/state discourses and local perspectives leading to failure of many of this type of planning schemes [9–11], it is equally important to reflect on the extent to which the governance strategy adopted by these programs, which emphasizes static steady state-based, blueprint-driven managerialist interventions, can effectively deal with the inevitably open socio-ecological dynamics, complexity, uncertainty, and non-linearity that underlie parasitic transmission in human communities [12–14].

The importance of understanding the central role of socio-ecological dynamics for the appropriate governance of social change has been highlighted by work carried out in governance studies [12, 13, 15–19]. These demonstrate how addressing issues of system complexity, *viz.* that socio-ecological systems are not only complex, dynamic and dissipative, but are also adaptive and display uncertain emergent properties, need to be at the core of any attempt aiming to transform persistent societal problems. A major insight from this growing body of work is that accepting ecology-in-contexts and system uncertainty, including with respect to problem framing, means that there may be no singular path to disease control [18–20]. Instead, attention is drawn to a need to be open to the fact that there may well be multiple possible routes available for achieving successful disease control in different socio-ecological settings. Indeed, this body of work has contrasted how attempting to impose a rigid orderly outcome on an inherently complex adaptive system is often the cause of failure of such plans [13, 17]. This work has also shown how consideration of complex dynamics highlights the important need to shift to a different relationship between expertise and policy making, in which recognition of inclusive multiple framings of issues by all partners, including the local community, and reflexivity around such framings is essential to “open” up policy problems and options by addressing uncertainty, dissent, agency and controversy, rather than these be “closed down” by narrow visions of change, management, and progress [12, 17–23]. It has pointed to the need for an approach to policymaking and governance that is incremental and adaptive, based on deliberation, reflection and learning, and where political interests are brought explicitly into the picture. These insights challenge the current global governance of parasite control programmes, which true to its underlying instrumentalist vision of development [24–26], offers in this context only a limited, restricted vision of what is possible, excluding the context-driven outcomes of complex ecological dynamics and the socio-political processes of governance. These considerations underline that the basic Newtonian, normal science problem-solving paradigm that has underpinned and typifies most current parasite control initiatives has serious limitations when social issues and contexts form an integral part of the problem situation [13, 18, 20, 23, 27].

Here, our major focus is on how best to guide the management of the control or elimination of NTDs, given the context of the inherently complex, non-linear socio-ecological dynamics of parasite transmission in endemic populations. We begin by reviewing the impact of this complexity on NTD transmission, and how attention to complex system dynamics is a requirement for providing a useable guide to action. We then discuss the policy, organizational and management responses that have been proposed to deal with dynamic socio-technical-ecological problems, and highlight governance frameworks that explicitly address notions of system complexity, non-linearity, uncertainty, and resilience. We next contrast this with the current global governance of NTD control, arguing for the need to develop a more inclusive and reflexive governance model that provides scope for linking local social contingencies and priorities with disease control policy. We examine recent work in the governance of complex socio-technical change to draw out a practical organizational framework that would allow the balancing of the institutional flexibility and stability required to enact a similar system of governance for NTD control. We end by pointing to some examples of complexity-based management structures that have been applied successfully to parasite control, and to research needs for the development of similar structures that may better generate and manage sustainable pathways for accomplishing global NTD control effectively.

## Review

### Complex dynamics of NTD infections

**Fig. 1:**
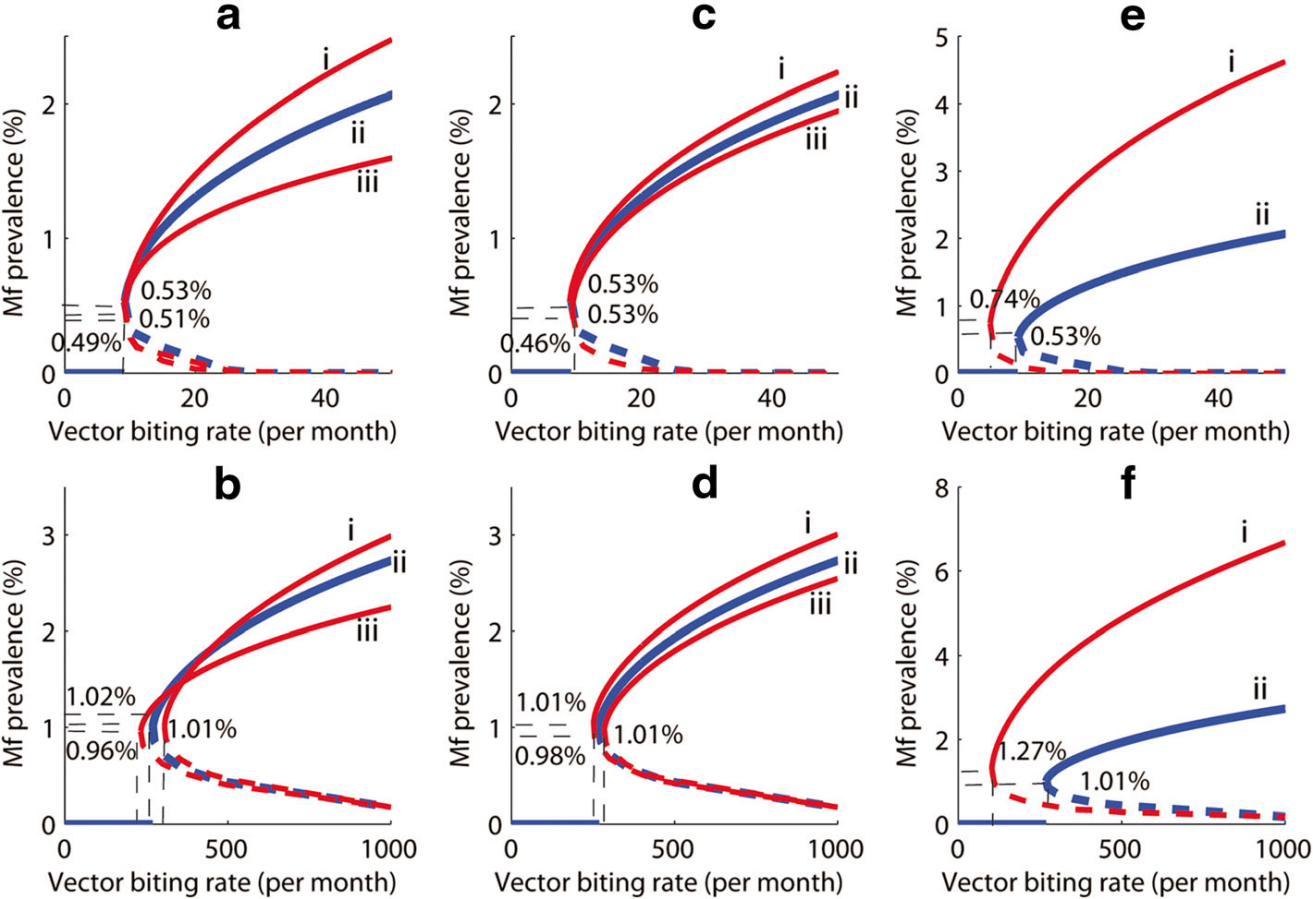
Sensitivity of the endemic microfilarial (Mf) infection prevalence breakpoint values and threshold vector biting rates to changes in the shape parameter of the negative binomial distribution, (*k*) describing the distribution of mf among human hosts (**a** and **b**), the strength of the immune response to infection, *β* (**c** and **d**), and per capita worm fecundity, *α* (**e** and **f**). The upper panel of graphs are for culicine-mediated filariasis, whereas the graphs belonging to the lower panel represent simulation results for anopheline filariasis. When the intermediate host is culicine, the results indicate that the maximum value of the mf breakpoint (*values on Y-axes for all graphs*) can change with *k*, but the vector biting thresholds (*units are bites per month on X-axes for all graphs*) will not change (TBR = 9) (**a**). For anopheline intermediate hosts (**b**), both the maximum mf breakpoint and the TBR increased with increasing *k* (curves iii to i). The parameter *β* can be thought of as an index governing the strength of host immunity in reducing establishment of parasites in the human hosts. The *β* values were varied by 10% so that the values used here were: i) 0.1, ii) 0.112, and iii) 0.123. When the intermediate host is culicine (**c**), the maximum value of the mf breakpoint changed with *β*; but the TBR does not change from its value of 9. For anopheline intermediate hosts (**d**), the maximum mf breakpoint increased and the TBR decreased with decreasing *β*. Graphs **e** and **f** depict the mf prevalence values found for values of per capita worm fecundity, *α*, of either i) 0.4 or ii) 0.2. Whether the intermediate host is **e** culicine or **f** anopheline, the maximum value of the mf breakpoint rises and the TBR lowered with increasing *α*. Results as per Gambhir & Michael [34]

Ecologists have long recognized how non-linear interactions in even very simple systems can result in highly dynamic behavior and patterns over time [28–30]. Recent studies have underlined how such dynamics, characterized by complexity and uncertainty, are also the norm for almost all social, ecological and biological systems [14, 18, 22, 31, 32]. We have recently demonstrated how these dynamics could typify the emergence, transmission, and extinction dynamics of parasites causing NTDs [14, 33–37]. Our mathematical studies of lymphatic filariasis (LF) – one of the major NTDs chosen for global elimination by the World Health Organization (WHO) – have shown, for example, how transmission and infection breakpoints, the threshold variables that govern the switching of this vector-borne helminth system between a stable endemic and an extinction steady state, are highly variable within- and between communities as a result of both site-specific variability in the ecological, biological and socio-economic variables that underlie parasite transmission, as well as the stochasticity and uncertainty surrounding the values of these drivers of transmission (Fig. 1).

**Fig. 2:**
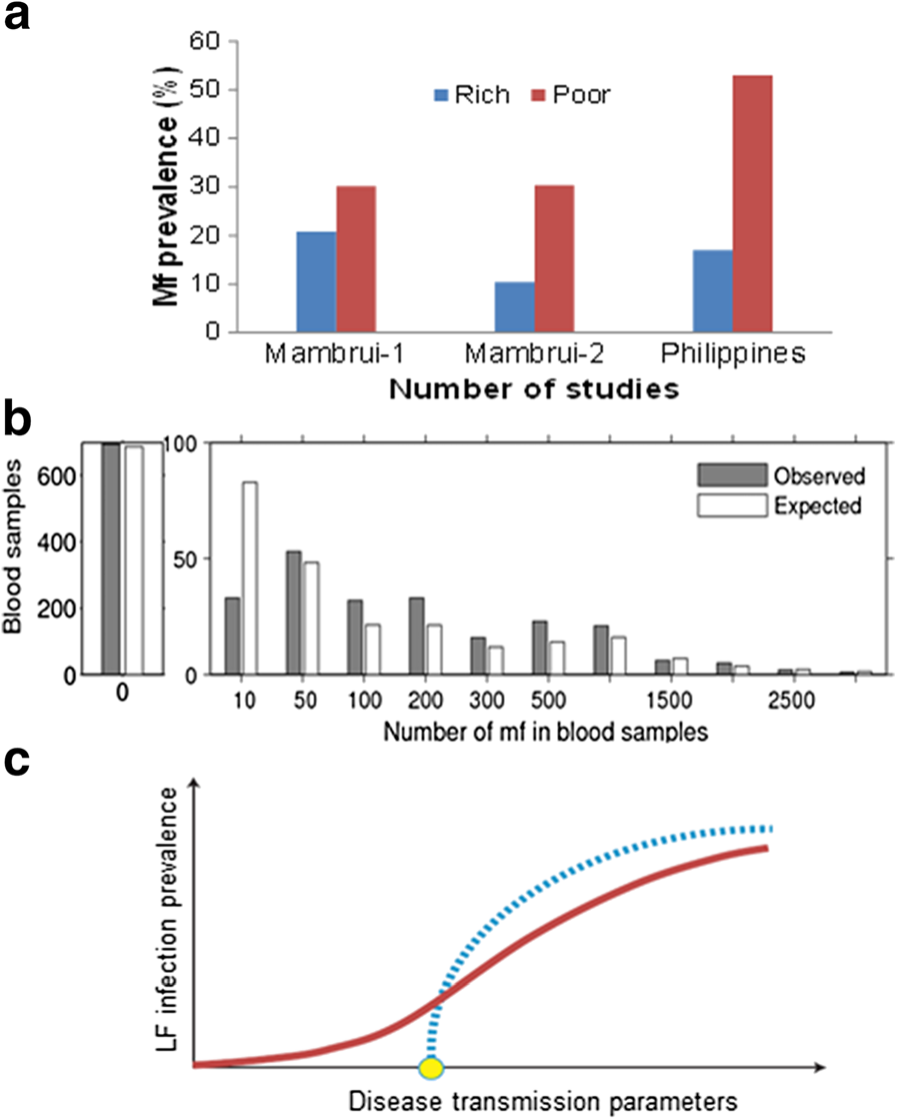
The impact of the socio-economic factor of poverty on the distribution of lymphatic filariasis (LF) infection and extinction thresholds. **a** Prevalence of LF Mf prevalence is disproportionately aggregated in the poor (*p* < 0.05). Data for Mambrui from Wijers and Kinyanjui [67]; and for Philippines from Grove et al. [68]. **b** The typical distribution of LF infection in a community. Estimation of the aggregation parameter *k* from the observed Mf count data indicates a highly over-dispersed/heterogeneous distribution and transmission of LF-mf in individuals of Masaika in Tanzania (data summarized in [69]; methods for estimating *k* and deriving the negative binomial frequency distribution are as given in [70, 71]). **c** Elimination thresholds for LF prevalence can vanish under heterogeneous/aggregated transmission conditions (*ie*. for low *k* values: *solid line*) compared to more homogenous transmission settings (with higher *k* values: *dashed line*). Methods used to estimate the transmission thresholds shown in the fig. as a function of *k* are as detailed in Singh et al. [37]

The finding that the pattern of infection aggregation in communities and the level of acquired herd immunity, both of which are linked to the socio-economic status of individuals, can act as critical drivers of the observed site-to-site variability of these transmission thresholds (Fig. 1a-d), in this connection, underlines the key importance of social factors in underlying the transmission dynamics of this parasitic disease in human communities. The specific impacts of such factors on LF transmission and extinction dynamics are outlined in Fig. 2, which show how: 1) a poverty-generated disparate distribution of parasite infection among individuals (Fig. 2a), may lead to 2) an over-dispersed or an highly aggregated distribution of infection within a community (Fig. 2b), which in turn 3) can have a major effect on values of community transmission thresholds, including in the extreme case of very high degrees of aggregation to reduction of these values to near “0” (Fig. 2c).

Another finding which characterizes the complexity of parasitic systems is the irreducible uncertainty connected with predicting transmission dynamics; this is underlined by the results depicted in Fig. 1e, f, which show how values of the microfilarial (Mf) production rate of adult female worms can significantly impact the values of transmission breakpoints. Given that the values of this function can never be precisely known, this fact together with the difficulty of measuring the local socio-ecological parameters (infection aggregation, acquired immunity), imply that the core challenge in eliminating complex dynamical diseases, such as LF, is fundamentally related to the problem of how best to enact strategies in the face of limits to knowledge and prediction of transmission and extinction dynamics in different local dynamic socio-ecological contexts. These observations indicate that the current top-down deterministic, command and control managerial practices, characterized by the belief that parasite populations should fundamentally respond to interventions similarly everywhere, and that technology and hierarchical health systems are the only and best forms of health delivery, are unlikely to achieve the goal of successful NTD control/elimination in all regions. Research from other complex dynamical contexts such as ecosystem or natural resource management indicates that the uncritical application of such linear, order-imposing, management models to complex problems may constitute the major source of the often unintended or pathological consequences that arise from societal interventions [38].

**Fig. 3:**
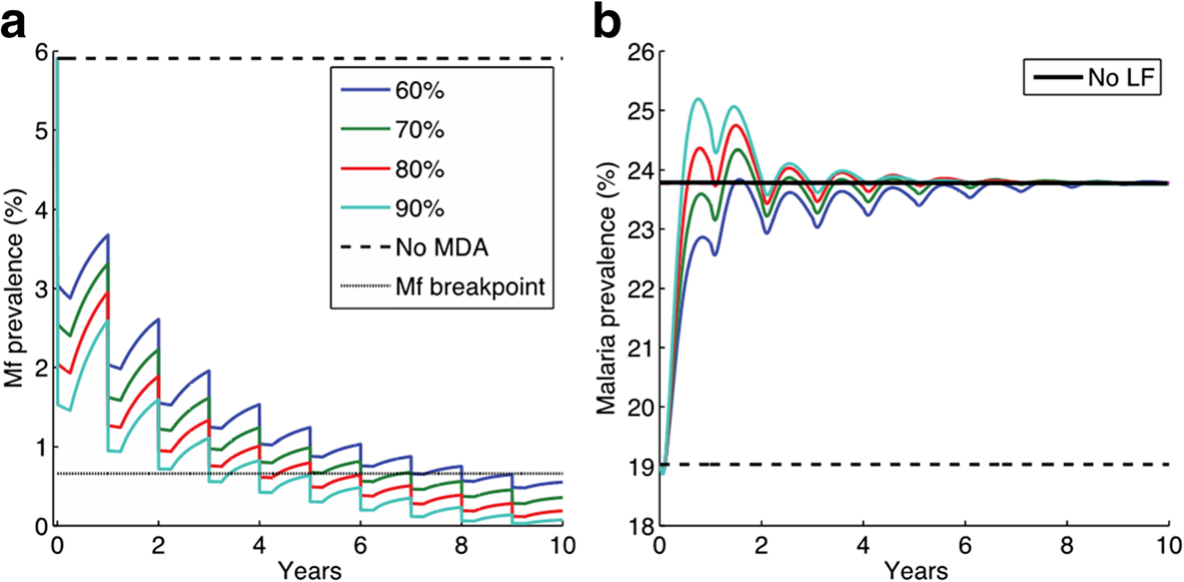
Results from a malaria-filariasis (LF) co-infection model [40] portraying the dynamic impact on each infection as a result of a 10-year annual MDA programme against LF in a community exposed to co-transmission of both infections by the same *Anopheles* mosquitoes. **a** Mf prevalence changes over time, and **b** the corresponding changes in malaria prevalence in the co-endemic community due to LF MDA. The predictions show that as a result of density dependent effects by LF larvae on mosquito survival, removal of worms by MDA (provided annually for 10 years) could give rise to a flare up of malaria cases in such co-endemic communities

Figure 3 depicts a cautionary example of this potential for unintended consequences that could arise from the application of analytical assumptions of equilibrium (rather than dynamic) thinking, centered on linearity, predictability, invariance, and simplification, as practiced by the current NTD control strategy. The simulation results in the figure portray how interventions that limit their target to one disease (*eg.* MDA against LF) may enhance the transmission of a more deadly disease, *viz.* malaria in this case, in those areas where both diseases are co-transmitted by the same vector mosquitoes [39, 40]. If it arises, this possible pathological outcome would resemble the devastating unintended contribution of mass treatments against schistosomiasis using emetin tartrate injections to the emergence and ongoing epidemic of Hepatitis C infection in Egypt [41]. These findings endorse the need to cater for perspectives based on holistic (rather than reductive) analysis, acknowledgement of the complexities of the ecologic and social context and their impact on the content of interventions, and the inevitability of uncertainty and limits to knowledge even in the most deterministic of systems, if more viable NTD control is to be achieved.

**Fig. 4:**
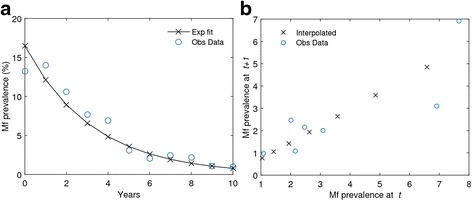
Characteristic changes in LF dynamics as infection approaches a critical transition point. **a** Model fits (*lines with crosses*) to Mf prevalence time series data (*open circles*) following a MDA intervention. **b** System slowness as it nears a transition, as evidenced by an increasing lag-1 correlation between subsequent states [30, 42]. Open circles: observed data; crosses: model predictions. Observed data are from Ramaiah et al. [72]; simulation methods used to model the impact of MDA on progression of mf infection towards a site-specific breakpoint threshold as provided in Michael and Singh [14]

A third emergent property of complex dynamical systems of significance to NTD control relates to the widespread finding that as a complex system, irrespective of whether it is natural or man-made, is pushed towards a critical threshold (e.g. infection breakpoint), it becomes increasingly slow in recovering from small perturbations [30, 34]. It has been shown that such ‘critical slowing down (CSD)’ in the case of natural systems nearing bifurcation or tipping points can serve as markers of impending system change to a new stable regime [42–45], destabilization of populations into cyclical, quasi-cyclical or chaotic states, and extinction [45]. Typically, signs of such critical regimes or an approaching transition to a different system state are characterized by: 1) declines in resilience of equilibria, 2) increased autocorrelation between states at subsequent time intervals, 3) increased variance, and 4) a possible flickering of states between alternate attractors in the vicinity of the unstable threshold points [30, 42, 44, 45]. Figure 4 provides empirical and theoretical evidence that such critical transitional behavior could occur in LF and most likely also in the case of other NTDs, suggesting that as a result of nonlinear feedback loops, multiple interacting components, and “sloppy” parameter combinations [14, 42, 43, 46], parasitic systems will tend to stay or “stick” in the region of the unstable point for relatively long periods of time, as they attempt to adapt and respond to the changing fitness landscape or circumstance induced by an intervention perturbation. This would not only lead to the requirement of maintaining unfeasibly long interventions, but also a need to accommodate heterogeneous adaptive responses across different localities. These results can also be interpreted by thermodynamics-like concepts which dictate that living systems resist the move to disorder and decay by the complexity of their internal structure and the continual use of energy and adaptive action to remain in or maintain a stable state of survival and, eventually, evolution [13]. The upshot is clear: a biological system by natural design will likely have some elements at all times that are able to fit to changing circumstances in order to ensure that the system as a whole can remain viable for long enough to adapt. Interventions aiming to disrupt parasite transmission must therefore guarantee the crossing of transition thresholds before these adaptations (e.g. by genetic rescue generating mutant resistant strains) take place, indicating not only that such parasite-control arms races will be an inevitable consequence of any chemical-based intervention attempt, but also that the consequent control dynamics are likely to be complex and even unpredictable.

### Governance responses to the management of complex social–dynamical systems

It is now widely recognized that social issues, whether arising in the health and disease, food and agriculture, natural resource and environment, or even within socio-economic /political domains, inevitably represent complex dynamical problems, and that given such system complexity the rational planning and institutional frameworks used traditionally are particularly ill-suited to their effective management [15–19, 47]. By contrast, increasing recognition that solutions for these problems depend a great deal on the development of governance structures that allow for understanding of the problem situation within its context and the efficient addressing of system complexity and uncertainty underpins the alternative governance and management paradigms emerging in the twenty-first century (Table 1). In terms of system management, the core question in this type of governance may be identified as relating to how best to understand and manage the dynamics or trajectories of the system to achieve a beneficial objective rather than implementing interventions assuming that the system is characterized by stability and equilibrium, i.e. contain a clear common, controllable, end point target [13, 18–20]. Thus, the analytical focus of these newer so-called “post-normal science” paradigms is placed more on understanding and learning regarding the processes of change, and on how governance arrangements can be developed which are best suited to cope with and adapt to a dynamic and constantly changing environment without universal structures or end points, rather than those assuming that these features, and the solutions for their control, are well known and are predictable [18, 48, 49].

**Table 1:** Contrasting paradigms of governance of the twentieth century and an alternative paradigm emerging in the twenty-first century

Dominant paradigm displayed in the twentieth century	Emerging alternative paradigms for the twenty-fist century
Problem solving/goal seeking orientation	Learning orientation
Focus on short term gains	Focus on sustainability and the long-term
Assumed predictability and certainty	Unpredictability and uncertainty
Control	Adaptive/reflexive/transitional management
Linear causality	Recognition for holistic/integrative thinking
One “truth” or best answer	No final “best” answer
Observer status objective	Observer status is constructed/subjective
Focuses on parts	Focuses on the whole
Analysis/reduction	Synthesis
Structural constancy	Structural changes affect function
Asymptotic stability	Multiple stable states
Reliance on simple cause and effect	Recognition of emergent properties of systems
Assumes systems models to be models of the world (ontologies)	Assumes systems models to be intellectual constructs (epistemologies)
Science and technology have the required answers	Scepticism and critical evaluation of science and technology
Talks the language of problems and solutions	Talks the language of “situation of concern” accommodations

Adapted from: Allison & Hobbs [20]

These new approaches to governance of social-technical-ecological systems also acknowledge the important roles that human agency and reflexivity play in a problem formulation and its solution, the significance of understanding how ‘governance’ shapes the direction of scientific and technological processes, including the framing and addressing of social issues, and how intervention consequences become distributed and indeed whether these are acceptable to all [12, 15–17, 20, 50]. Therefore, a second key development in modern governance arrangements has been the evolution of structures that allow for diversification of actors and institutions in policy making to enable the “opening up” of a broader analytical and political agenda that addresses the multiple processes and relationships through which the state and a wide range of non-state actors might engage in steering recognition as well as development of acceptable solutions to persistent social problems. The idea behind this evolution of modern governance systems from the traditional rule-based, hierarchical, top-down Weberian-type command and control state-led governance approaches into forms, such as networked governance, public-private partnerships, and adaptive multi-tiered and multi-actor/stakeholder governance, is that incorporation of such diversity of interests into the policy and governance process is not only thought to represent a more effective way to cope with complexity and incertitude, but it is also believed to produce a more flexible and responsive governance process for solving complex social problems [17–20, 50, 51]. The development of newer forms of reflexive and transition governance or management structures, which emphasize combinations of stakeholder involvement, trial-and –error policy experiments, and context-specific but network-based adaptive policy making, is in this regard an attempt to take a fuller account of dynamic and complex system responses in order to develop measures to steer rather than control system dynamics to negotiated ends [17–19, 21, 50]. However, note that while a diversification of actors may increase the range of values and perspectives to be brought into the policy arena, power imbalances (both in terms of financial and knowledge disparities) between actors mean that these approaches can also be considered as neoliberal strategies that aim to implement non-state-led solutions based on self-interest and commercially-driven incentives to reach social goals, as has been noted to constitute an emerging problem with current health policy in developing country settings [5–7].

### Contemporary governance and management of NTD control

**Fig. 5:**
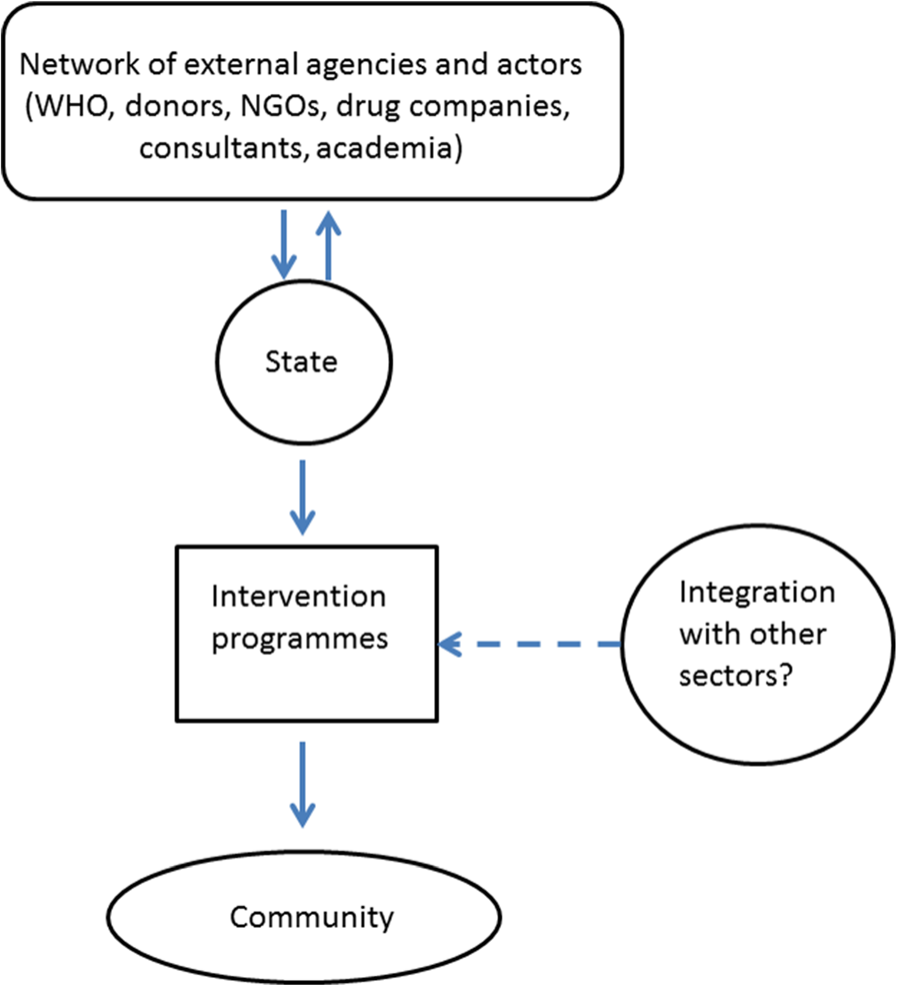
Governance structure and institutions underpinning the current global NTD control programme

Figure 5 provides a broad outline of the major components of the prevailing governance structures and institutional frameworks underlying the current global NTD control initiative. Three features are immediately apparent. First, in line with the emerging alternative paradigms of governance as described above, the figure shows that policy setting, and intervention formulation and design, indeed do take place among a network of diverse global and national institutions and partners (Fig. 5). While this can be considered as a mechanism for allowing a broadening of multi-actor and multi-level perspectives and solutions, the focus remains on a narrow intervention agenda based primarily on MDA plus the mostly unidirectional (with some feedback from states, but not from communities) flow of knowledge and funds (denoted by arrows in the figure (see Madon et al. [52])). As a result, such networks in reality form globally interrelated “epistemic communities” of like-minded actors and institutions which has the effect of narrowing down problem definition and framing, and producing selective knowledge and solutions [5, 7, 12]. Studies have highlighted that a key feature of such closed networked systems is how decision-making is led by accredited forms of expertise that help construct issues in a certain way and develop measures to act on them to produce particular material effects. Governance and policy processes are thus appropriately “evidence-based”, with evidence often constituted in relation to a singular notion of “sound science”, and any contestation over knowledge excluded from the policy discourse. In the context of current power and knowledge relations between the north and south, another drawback, as has been pointed out by several authors, is a predilection for the domination and capture of policy making on global issues by the powerful north [5–7].

While some argue that such networks, by promoting shared problem-framings amongst people and institutions across globally-connected spaces, are an important means for placing issues of the marginalized on state agendas as well as for exerting moral pressure on governments to act [1, 4, 8], this needs to be balanced against several less positive outcomes. First, as noted above, problem definitions and solutions are largely driven by external global discourses leading to a hollowing out of the state and thus locally-relevant or context-specific solutions. Indeed, in this governance heuristic, the state is conceived simply as the implementing agency of policies developed largely by external actors/institutions. Paradoxically, thus while there is an attempt to open up policy making at the highest level of the hierarchy, the state is fundamentally seen as leading the delivery of what are essentially prescriptive, top-down solutions [53]. While this shift can be considered as an example of a “hollowing out” of the state by leaching power away vertically [51], it is also instructive to note how such a hollowing out could be orchestrated by the state to work in favour of sustained, even enhanced state control [51, 54]. In this view, the state “manages” the risk of overload and failure by devolving some activities and powers, but retains key influence over policy agendas and strategies. A key result of this “crowding out” or hollowing out of regional planning contexts and perspectives, however, is a lack of knowledge and consideration of different preferences for public goods, which can impact local uptake and compliance with proposed interventions, including giving rise to conflicts regarding potential benefits at multiple scales and between different actors [8–11]. A growing body of anthropological work shows how such resistance to external disease control programmes may be closely linked to local understandings of disease, relationships to health and government officials, and past experiences with development projects [8]. Second, as noted by many authors, the cultivation of “epistemic” networks of practitioners that share a common philosophical and theoretical mental construct in policy making also leads to the discipline-bounded vertical solutions typically observed in global health delivery. These solutions have been instrumental in the increasing fragmentization and “projectification’ of health care focused on specific diseases [6, 7], and the development of standardized, often pharmaceutical-driven biomedical interventions, based on perspectives emphasizing individual risk factors rather than the social determinants underlying health outcomes (*eg.* poverty, inequity, lack of development [5–8]. Finally, the approach reinforces the normal science technicist, managerial, command and control-type interventions with its attendant assumptions that preferences and solutions are well known and established, and a downplaying of system complexity and incertitude.

## A complexity-based governance structure for NTD control

The preceding sections indicate that parasite control may represent a complex problem with uncertainty, non-linear dynamics and social involvement comprising the predominant characteristics, and that as such they are likely to defy reduction and empirical testing by the traditional normal science paradigm. These characteristics also underline the increasing recognition that the failure of science to resolve certain trenchant social problems may be due to uncertainty about whether the appropriate questions are being asked, and whether problems are addressed with the relevant theoretical and methodological tools and within an appropriate paradigm [15, 16, 20, 48]. These features indicate that the key components of a complexity-based governance framework for NTD- and indeed other parasitic diseases – control must, at the very minimum, include the means to reliably meet three major needs. First, structures and processes are clearly required that can assist with the inclusion of diverse perspectives. Second, development of approaches to enhance institutional learning are needed at all levels for both reflexively enquiring into and resolving the process by which values are constructed and used in making public choices so that these can be adapted according to local contexts and development needs. And finally, methods are required to institute recognition and accommodation of the effects of emergent system properties, i.e. that these are likely to produce unpredictable and uncertain future system behavior. Thus, the main objective of management in this regard should be to implement interventions that will facilitate steering of the parasite system towards a desired state while having the flexibility and adaptive capacity to cope with the inevitable surprise and unpredictability that will arise along such pathways [18, 38].

These are not merely theoretical arguments for a need for change in current NTD governance arrangements; this requirement is also underscored by the outcomes of previous global disease control/eradication governance configurations. Thus, while the global smallpox eradication began initially by rolling out a highly prescriptive approach (based on a simple and cheap technology blueprinted, designed, and specified at the top and to be implemented uniformly by rote at the bottom), it had to be replaced by a more incremental and adaptive implementation approach that even led to a complete revision of the original faulty assumptions (80% vaccination coverage applied uniformly everywhere was adequate for elimination, for example) and derivation of locality specific implementation strategies that had to mesh with health system deficiencies, difficult terrain, climate, and cultural beliefs (see Henderson, D. 1999: http://www.cdc.gov/mmWR/preview/mmwrhtml/su48a6.htm). Similarly, the history of the Global Malaria Eradication Programme (1955–1969) supports the same conclusion, *viz.* that a heavily top-down prescriptive approach based on simple technological tools will more than likely fail. Lessons from that failure highlight the fact that no single strategy can be applicable everywhere and that a long-term commitment with a flexible strategy that includes both active community involvement and integration with the local health system will be needed for successful parasite control [13, 55–57]. By contrast, examples of the governance of the soil-transmitted helminth, schistosomiasis and lymphatic filariasis control programmes implemented successfully in China and Japan point to how construction and application of a pluralistic framework based on: 1) local policy development accompanied by strong political commitment, 2) use of multiple interventions in an integrated fashion through intersectoral action, 3) adaptation of control options for specific eco-epidemiological settings and over time as the challenge of control changes, 4) active participation of the community and other stakeholders (both private and public), and 5) formation of strong linkages to research and learning activities using surveillance and monitoring data [58–60], will be important if we are also to similarly accomplish global NTD control.

What might the practical form of a complexity-based NTD governance organization look like? Recent work on governance structures better equipped to address and manage processes characterized by complex change suggests that this task technically requires addressing both institutional stability and change [15, 16], as well as the bridging of multilevel linkages [17, 18, 22, 23]. In this view, institutional stability is necessary for accumulating resources, including those needed for coordinating and implementing cooperative programmes, but at the same time, organizations need to be flexible, i.e. able to change in order to adapt to novel circumstances. It is thus thought that key to the establishment of these institutional structures is the efficient resolution or management of the tension that exists between the need to achieve “explorative” activities with the “exploitative” capacity of institutions, which contrastingly requires stable structures (and so some rigidity) [20, 61]. Accordingly, these practitioners have highlighted how constructs from organizational theory can be used to design or retrofit such a governance structure to enhance complexity-handling performance.

**Fig. 6:**
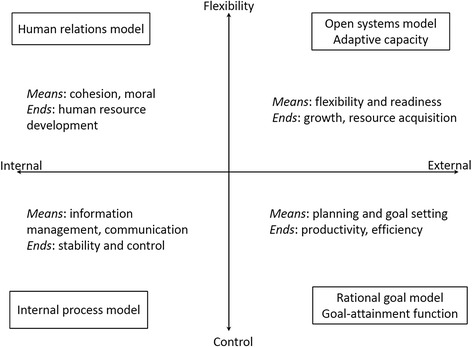
The two-dimensional organizational effectiveness framework as a template for developing complexity-based NTD governance structures. Adapted from Quinn and Rohrbaugh [62]

Figure 6 is an attempt to apply insights from organizational theory to guide the development of a similar adaptive complexity-based governance model for NTD control, showing the different means and ends to achieve the resolution of the stability-flexibility or exploitation-exploration tension both internally and externally. Here, drawing on the work of Quinn and Rohrbaugh [62], organization effectiveness in blending the tension between the static and dynamic are arrayed along two spatial dimensions. The first dimension, represented on the horizontal axis, is related to organization focus, either from an internal or an external emphasis on the development of human resources in the organization itself. The second dimension, represented on the vertical axis, differentiates between dichotomies in structure within an organization, identifying either a preference for stability and control, or a preference for flexibility and change. Each quadrant of the framework represents one of four major models of organization and management theory. Thus, the human relations model places emphasis on flexibility and an internal focus, stressing human resources development as criteria for effectiveness. The open systems model, by contrast, emphasizes flexibility and external focus, stressing the organization’s readiness, growth, resource acquisition and external support. The rational goal model in turn emphasizes control and an external focus, and views planning, goal setting, productivity and efficiency as effective. Finally, the internal process model emphasizes control and an internal focus, stressing the role of information management, communication, stability and control. Such a framework clearly allows the operation of the organization through the phases required to better address and manage processes and responses characterized by complex change, *viz.* the unpredictable outcomes arising from uncertainty, nonlinearity, and adaptive dynamics. It is also dynamic, with the focus for the organization at times swinging towards the process or the means, and at other times towards the ends or the goals [62].

## Future work and governance templates

Additional questions that need resolving for developing such a system would touch upon how best to create institutional rules and mechanisms to ensure incorporation of all perspectives into and cooperation among all actors in the NTD global-regional governance system, so that efficient discovery of methods and delivery of NTD control to endemic communities is made possible. This may also include establishment of means to leverage the existing power relations among dominant local/external stakeholders who direct current agendas and discourse in global NTD control so that parasite control can be delivered in a more locally-acceptable manner. Such processes will therefore also need to include structural forms best able to align preferences of citizens in the provision of control, including the means for making choices that give citizens a voice in collective decision making. Key needs here may include ways of how best to promote recognition by individuals of their interdependencies and their dependence on the global community to address certain issues, and the forms of incentive schemes that will lead to transformation of beliefs so that agents at various levels can internalize collective issues in their own system of preferences. The proposed establishment of village health committees in Tanzania identified by the government as an entry point for promoting community engagement in NTD control may be seen in this regard as an organizational approach to achieve this goal, particularly in terms of aligning interventions with local priorities and social norms [63].

A second question related to how best to enhance the explorative abilities of institutions at different levels engaged with NTD control, would be the derivation of management structures focused on nurturing learning and undertaking experiments, and the ability to respond reflexively and effectively to complex adaptive change over time. This also needs to include the means to address the politics of knowledge production for guiding generation of alternative local knowledge more effectively, and ensuring its diffusion so that stakeholders at all levels can make informed collective assessments, preferences and decisions better. An important requirement connected with learning and reflexivity is related to creating the institutional capacity for analyzing and explicating clearly the paradigms, philosophies and assumptions that underpin both chosen and proposed approaches to problem setting and solutions. Such explicit expositions will be critical for not only assessing the effectiveness of proposed methods for achieving sustainable NTD control but also for investigating how biases in thinking and action affect how programmes are evaluated and judged as successful or as failures [12, 18, 20, 27]. This conclusion echoes similar arguments made by Marchal et al. [64] for the importance of increasing capacity-building at all levels of the health administration for experimenting and learning about how NTD campaigns and general health systems interact.

Finally, there is a need to improve recognition at all levels that knowledge of the future is made difficult due to the uncertainty of future system dynamics. This means that there is a need to enhance management and scientific capacity to: 1) use monitoring data within a framework of multiple perspectives, values and needs, and 2) support adaptation of interventions as information grows so that rather than aiming for a set “optimized” solution, which assumes certainty in outcomes and hence linear cause-effect controllability, hypotheses and options are generated, tested and explored over time and under different socio-ecological contexts to steer an infection system towards effective, long-term control. It is interesting to note, in this context, that Madon et al. [52] found that village leaders in Tanzania have the potential and willingness to become involved with local monitoring of NTD control programs and to help find local solutions to reduce the burden of disease. This suggests that exploitable capacity may exist even at the local level for developing the type of learning frameworks required for searching and implementing bottom-up adaptive parasite control solutions in NTD endemic countries.

There are currently few examples of national NTD control programmes that have elements of these organizational requirements formally built into their management structures. However, the examples from China and Japan alluded to above indicate that specific structures that can accommodate an inclusive, pluralistic design to facilitate diverse perspectives from different stakeholders, are flexible and adaptive to the choice of interventions, and have learning included into policy and programme development, will be important. The exact organizational structures used in these countries are not well known, but work by Spiegel et al. [65] on how intersectoral action for health (IAH) programmes are implemented in Cuba at the municipality level offer a glimpse of the type of organizational structures that could serve as a template for developing a complexity-based NTD intervention programme. The key institutional innovation here is the Municipal Health Council linked to the health system that has representatives from various population sections, including the community, different sectors of the state, the local political party, and entrepreneurs, for serving as a forum to deliberate, adapt, and formulate local plans and activities for implementing agreed upon national health programmes [65]. Another example is afforded by the Nepal Urban Ecosystem Health Project, wherein on finding that a purely epidemiological approach to echinococcus disease control was not sufficient to interrupt the transmission cycle, participatory approaches to engage relevant stakeholders in mutual learning and behavioral change – based on a negotiated vision and action plan, were enacted and found to be more effective [66]. The key institutional changes made were: 1) creation of a decision space for ward officials and different community stakeholders to come together to work on practical initiatives to improve community life (but with echinococcus disease control forming one focus), 2) development of local “clubs” that took on specific tasks like organizing recycling drives and garbage pick-up, and 3) mobilization of support for on-the-ground action via organization of grass roots meetings with people from the community to analyze, resolve competing interests, and search for and act on feasible locally-acceptable solutions (https://www.idrc.ca/en/article/health-urban-environments). It is instructive here to note how incorporating community interests may require programmes to refocus disease-specific goals to also address the under-lying structural drivers of parasitic disease transmission (to include garbage collection and urban reneval activities, in this case), which could in turn help build long-term societal resilience to disease re-emergence [8, 47]. We indicate that social and organizational research into the configuration of similar systems tailored to different social settings, and how they may be practically constructed, implemented, sustained and evaluated, is now critically required to open up the debate on appropriate governance structures for undertaking parasite control in response to the complexity, variability and change typically representative of highly dynamic contexts.

## Concluding remarks

Our discussion indicates that a more sustainable approach to NTD control critically requires a joining together of non-equilibrium thinking about the operation of complex systems from the natural sciences with social science perspectives to allow development of governance structures better designed to cope with the uncertain socio-ecology of NTD transmission and control. We have outlined and discussed an analytical framework that could be used to develop a governance process and structure that allows integration of these approaches for enabling the goal of achieving sustainable parasite control. What makes such an approach different from the traditional, positivist, reductionist, managerial, forms of governance is the focus on clarifying the strong link between content and process, and the emphasis placed on the importance of being open to policy searching, learning, deliberation and experimentation, while simultaneously having stable institutional components focused on achieving “exploitative” functions. It is founded on understanding the dynamics of complex, adaptive, socio-ecological systems, and how such understanding provides insights into the opportunities, limitations and conditions under which it is possible to steer or direct such systems in a collective, inclusive, manner. The justification for taking account of system complexity, review of the governance literatures, and the organizational analysis presented in this paper present a first attempt to point a way forward in this challenging but vitally important topic, not only in the context of NTD control but also with regard to the effective control of other major global diseases. Given the crucial role that social science methods play in clarifying the normative components and dimensions of such a complexity-based governance approach, this also means that social science research, including organizational and management science, must together with the natural sciences be at the heart, and not be pushed towards the periphery, of scholarship on NTD control. We echo Bardosh [8] in suggesting that the development of such coupled social and natural science scholarship is now critical given the increasing initiatives being developed for achieving the control of parasitic diseases globally.

## Additional file

Additional file 1: Multilingual abstracts in the six official working languages of the United Nations. (PDF 481 kb)

## Abbreviations

CSD: Critical slowing down
LF: Lymphatic filariasis
MDA: Mass drug administration
Mf: Microfilariae
NTD: Neglected tropical disease
WHO: World Health Organization
