# Supplementary material for: Socio-ecological dynamics and challenges to the governance of Neglected Tropical Disease control
Source: Infect Dis Poverty. 2017 Feb 6;6:35. doi: 10.1186/s40249-016-0235-5 (PMC5292817; doi:10.1186/s40249-016-0235-5)
Supplement: Additional file 1: — Multilingual abstracts in the six official working languages of the United Nations. (PDF 481 kb) [file 40249_2016_235_MOESM1_ESM.pdf]

Translation of the abstract into the six official working languages of the United Nations

الديناميكيات الإجتماعية- البيئية و التحديات التي تواجه إدارة مكافحة الأمراض المدارية المهملة.

إدوين مايكل و شيرين مادون.

ملخص.

إن الجهود العالمية الحالية لمكافحة ما يُدعى "الأمراض المدارية المهملة" لديها القدرة بشكل كبير على الحد من الأمراض التي يعاني منها بعض أفقر المجتمعات في العالم. ومع هذا، فإن إدارة برامج المكافحة هذه مدفوعة بالسلوك العقلاني الإداري الذي يفترض إمكانية التنبؤ بالتدخلات المقترحة، و التي تسعى في المقام الأول إلى تعزيز فعالية تكاليف التنفيذ عن طريق قياس الفعالية من حيث النتائج المحددة سابقاً. هنا، نحن نرى أن هذا النهج قد عزز النموذج المحدود للعلم الطبيعي لمكافحة الأمراض الطفيلية، و بهذا يكون قد فشل في معالجة الديناميكيات المعقدة، و عدمية اليقين وخصوصية السياق البيئي الاجتماعي الذي يكمن وراء عملية انتقال الطفيليات. نحن نقترح أننا بحاجة إلى نهج إداري جديد يعتمد على التفكير اللاتوازني حول الأنظمة المعقدة و التكيفيّة من وجهة نظر العلوم الطبيعية و العلوم الإجتماعية البنائية التي ترى تراكم المعرفة العلمية متوقف على الإهتمامات و المعايير التاريخية في حال تم ابتكار طرق مكافحة فعالة ذات حساسية لسياق الأمراض المحلية.

في صلب هذا النهج هناك تأكيد على الحاجة إلى عملية تساهم في إدراج وجهات نظر متنوعة، التعلم الإجتماعي و التداول، النهج الإنعكاسي لمعالجة تعقيدات و تذبذب النظام وفي نفس الوقت تحقيق هذه المرونة بهيكلية تسعى للتوازن و الإستقرار. نحن نستمد و نناقش إطار عمل إداري محتمل و نحدد هيكل تنظيمي يمكن الإستفادة منه للتعامل بفعالية مع تعقيدات تحقيق مكافحة الأمراض المدارية المهملة NTD. نريد توجيه الأنظار أيضاً إلى أمثلة الهياكل الإدارية المبينة على مواجهة التعقيدات و التي طالما كانت تستخدم في مكافحة الطفيليات سابقاً و التي من الممكن ان تخدمنا كقوالب عملية لتطوير هياكل إدارية مشابهة لتحقيق إدارة عالمية أفضل في مكافحة الأمراض المدارية المزمنة NTD. نتائجنا تحمل تضمين هام وواسع لسياسة صحية عالمية تهدف إلى السيطرة و القضاء على الأمراض الطفيلية بفعالية حول العالم.

Translated from English version into Arabic by Rola Majbor, through

TRANSLATORS
WITHOUT BORDERS

管理被忽视热带病控制项目的社会生态动力学和挑战

Edwin Michael & Shirin Madon

摘要

目前，全球努力控制所谓的“被忽视的热带病（NTDs）”，可能使其在一些世界上最贫穷社区的发病率大大降低。然而，管理这些控制项目受被认为具有可预测性的干预措施的管理理性驱动，因此主要通过预定产出衡量绩效以提高项目实施的成本效益。我们认为，上述方法强化了控制寄生虫病的常规科学模型的限制性，且无法解决复杂的动态特征、不确定性和社会生态环境特异性，而这些始终是寄生虫传播的基础。我们认为，如果要设计、运用和管理更有效的且对当地疾病情况足够敏感的控制方法，就需要一种新的治理方案。它利用非平衡组合，考虑到自然科学复杂的、自适应系统的运作和认为科学知识的积累取决于历史利益和规范的建构主义的社会科学观点。此方法的核心是强调需要一个过程来帮助纳入各种观点、社会学习和审议，以及一种反映性方法来解决系统复杂性和不确定性，同时平衡这种灵活性和稳定性为重点的结构。我们推导和讨论了一个可行性治理框架，并概述了组织架构，可用于有效处理实现全球 NTD 控制的复杂性。我们还指出，在以前寄生虫控制中使用过的基于复杂问题的管理结构，可以作为开发类似结构的实用模板，以更好地管理全球 NTD 控制项目。本研究结果对全球卫生政策具有重要而广泛影响，旨在有效控制和根除世界各地寄生虫病。

Translated from English version into Chinese by Jin Chen, edited by Pin Yang

CHINESE CENTER FOR DISEASE CONTROL AND PREVENTION
NATIONAL INSTITUTE OF PARASITIC DISEASES

Dynamiques et défis socio-écologiques pour la gouvernance du contrôle des maladies tropicales négligées

Edwin Michael et Shirin Madon

Résumé

Les tentatives mondiales actuelles afin de contrôler les dénommées « maladies tropicales négligées » ont le potentiel de réduire considérablement le taux de mortalité dont souffrent les communautés les plus pauvres. Cependant, la gouvernance de ces programmes de contrôle est menée par une logique managériale qui suppose la prévisibilité des interventions proposées, et qui par conséquent, cherchent tout d'abord à optimiser la rentabilité de ces mises en œuvre en mesurant les performances en termes de rendements prédéterminés. Ainsi, nous affirmons que cette méthode a confirmé l'insuffisance du modèle de science normale pour contrôler les maladies parasitaires, et en faisant cela elle ne répond pas aux dynamiques complexes, à l'incertitude et au contexte socio-écologique spécifique lesquels sous-entendent systématiquement une transmission parasitaire. Nous suggérons qu'il est nécessaire de créer une nouvelle méthode de gouvernance qui mobiliserait un ensemble non-équilibré prenant en compte le fonctionnement des systèmes complexes et évolutifs provenant des sciences naturelles et du point de vue constructiviste des sciences sociales qui considèrent que l'accumulation des connaissances scientifiques dépendent des normes et des intérêts historiques, si seulement plus de méthodes de contrôle performantes et suffisamment réceptives aux contextes des maladies locales sont conçues, appliquées et maitrisées.

Au cœur de cette méthode nous mettons l'accent sur le besoin d'un procédé permettant la prise en compte de divers points de vue, de l'apprentissage social et des délibérations, ainsi que le besoin d'une méthode réfléchie pour répondre à la complexité et à l'incertitude du système, tout en compensant cette flexibilité par des structures stables. Nous en déduisons et évoquons un possible système de gouvernance et nous soulignons la possibilité d'une structure organisationnelle qui pourrait être utilisée pour véritablement faire face à la complexité du contrôle des maladies tropicales négligées au niveau mondial.

Nous mentionnons également pour exemples des structures de management basées sur la complexité qui ont été utilisées auparavant dans le contrôle des parasites, lesquels pourraient servir de modèles réalisables au développement de structures de gouvernance similaires afin de mieux gérer le contrôle mondial des maladies tropicales négligées. Nos résultats contiennent de vastes et importantes implications pour la politique de santé mondiale ayant pour but de contrôler et d'éradiquer de manière efficace les maladies parasitaires dans le monde.

Translated from English version into French by annesophi, through

TRANSLATORS
WITHOUT BORDERS

Социально-экологическая динамика и трудности организации борьбы с забытыми тропическими болезнями

Эдвин Майкл и Ширин Мэйдон (Edwin Michael & Shirin Madon)

Краткая аннотация

Глобальные попытки, предпринимаемые в настоящее время для борьбы с так называемыми «забытыми» тропическими болезнями (ЗТБ), могут потенциально значительно снизить смертность в некоторых самых бедных странах мира. Однако, организация таких программ строится на управленческой рациональности, которая предполагает предсказуемость предлагаемых мероприятий, и, которая, таким образом, в основном нацелена на минимизацию затрат при внедрении программ за счёт измерения эффективности, выражаемой в виде заранее установленных показателей. Здесь мы утверждаем, что такой подход укрепляет узкую модель нормальной науки борьбы с паразитарными болезнями и тем самым не в состоянии учитывать сложную динамику, неопределённость и социально-экологическую местную специфику, что всегда лежит в основе передачи паразитарных болезней. Если разрабатывать, внедрять и управлять при помощи более эффективных методов борьбы с болезнями, которые в достаточной степени учитывают местные условия, то мы считаем, что необходим новый подход, базирующийся на сочетании неравновесного продумывания функционирования комплексных адаптируемых систем естественных наук и конструктивистских социальных концепций, которые видят накопление научных знаний лишь в связи с историческими интересами и нормами. Основное внимание при таком подходе уделяется потребности в процессе, который способствует привлечению разнообразных взглядов, социальному обучению и обсуждению, а также рефлексивный метод решения проблем со сложностью и неопределённостью системы, при котором достигнут баланс между гибкостью и структурами, ориентированными на стабильность. Мы определяем и обсуждаем возможную схему управления и кратко описываем организационную структуру, которая могла бы эффективно справляться со сложностью задачи достижения глобальной борьбы с ЗТБ. Мы также приводим примеры нацеленных на сложность структур

управления, использовавшихся ранее для борьбы с паразитарными инфекциями, которые могут служить практическими образцами для создания аналогичных структур управления для улучшения глобальной борьбы с ЗТБ. Наши результаты имеют важные, более широкие последствия для глобальной политики в области здравоохранения, направленные на эффективную борьбу и ликвидацию паразитарных болезней во всем мире.

Translated from English version into Russian by Natalia Potashnik, through

Dinámicas y retos socioecológicos para la gestión del control de enfermedades tropicales desatendidas.

Edwin Michael y Shirin Madon

Resumen
Los intentos mundiales por controlar las denominadas “Enfermedades Tropicales Desatendidas (ETD)” tienen la capacidad de reducir significativamente la morbilidad que sufren algunas de las comunidades más pobres del mundo. Sin embargo, la administración de estos programas de control se hace siguiendo una lógica que asume la predictibilidad de las intervenciones propuestas y que busca primordialmente, mejorar la rentabilidad de la implementación midiendo el rendimiento en términos de resultados predeterminados. A través del presente documento, sostenemos que este enfoque consolida aún más el limitado modelo de ciencia normal para el control de las enfermedades parasitarias, y al hacerlo fracasa a la hora de tratar la compleja dinámica, incertidumbre y especificidad contextual socioecológica que subyace invariablemente a la transmisión de parásitos. Sugerimos que, si se van a elaborar, aplicar y gestionar enfoques de control más eficaces y lo suficientemente extensivos para los contextos de enfermedades locales, es necesario una nueva perspectiva en la administración basada en una combinación del pensamiento de no-equilibrio con respecto al funcionamiento de sistemas complejos y adaptativos de las ciencias naturales, y perspectivas constructivistas de la ciencia social que consideren la acumulación del conocimiento científico como dependiente de los intereses y las normas históricas. En el centro de este enfoque está el énfasis en la necesidad de un proceso que ayude con la inclusión de diversas perspectivas, aprendizaje social y deliberación, y un enfoque reflexivo para tratar la complejidad y la incertidumbre del sistema, al tiempo que se equilibra esta flexibilidad con las estructuras centradas en la estabilidad. Proponemos y discutimos un marco de administración posible y ponemos de manifiesto una estructura organizativa que se podría usar para tratar eficazmente la complejidad que supone conseguir el control mundial de las ETD. También mostramos ejemplos de estructuras de gestión basadas en la complejidad usadas previamente en el control de parásitos, las cuales podrían servir como modelos prácticos para desarrollar estructuras de administración similares para gestionar mejor el control mundial de ETD. Nuestros resultados acarrean importantes implicaciones de gran envergadura para las políticas sanitarias mundiales que tienen como objetivo controlar y erradicar de manera eficaz las enfermedades parasitarias en todo el mundo.

Translated from English version into Spanish by Barbara Gutierrez Teira, through
